# Theoretical aspects and modelling of cellular decision making, cell killing and information-processing in photodynamic therapy of cancer

**DOI:** 10.1186/1755-8794-6-S3-S3

**Published:** 2013-11-11

**Authors:** Ioannis Gkigkitzis

**Affiliations:** 1 Department of Mathematics, East Carolina University, 124 Austin Building, East Fifth Street, Greenville, NC, 27858-4353, USA; 2 Department of Biomedical Physics, C-209, Howell Science Complex, East Carolina University Tenth Street, Greenville, NC, 27858-4353, USA

## Abstract

**Background:**

The aim of this report is to provide a mathematical model of the mechanism for making binary fate decisions about cell death or survival, during and after Photodynamic Therapy (PDT) treatment, and to supply the logical design for this decision mechanism as an application of rate distortion theory to the biochemical processing of information by the physical system of a cell.

**Methods:**

Based on system biology models of the molecular interactions involved in the PDT processes previously established, and regarding a cellular decision-making system as a noisy communication channel, we use rate distortion theory to design a time dependent Blahut-Arimoto algorithm where the input is a stimulus vector composed of the time dependent concentrations of three PDT related cell death signaling molecules and the output is a cell fate decision. The molecular concentrations are determined by a group of rate equations. The basic steps are: initialize the probability of the cell fate decision, compute the conditional probability distribution that minimizes the mutual information between input and output, compute the cell probability of cell fate decision that minimizes the mutual information and repeat the last two steps until the probabilities converge. Advance to the next discrete time point and repeat the process.

**Results:**

Based on the model from communication theory described in this work, and assuming that the activation of the death signal processing occurs when any of the molecular stimulants increases higher than a predefined threshold (50% of the maximum concentrations), for 1800s of treatment, the cell undergoes necrosis within the first 30 minutes with probability range 90.0%-99.99% and in the case of repair/survival, it goes through apoptosis within 3-4 hours with probability range 90.00%-99.00%. Although, there is no experimental validation of the model at this moment, it reproduces some patterns of survival ratios of predicted experimental data.

**Conclusions:**

Analytical modeling based on cell death signaling molecules has been shown to be an independent and useful tool for prediction of cell surviving response to PDT. The model can be adjusted to provide important insights for cellular response to other treatments such as hyperthermia, and diseases such as neurodegeneration.

## Background

Conventional cancer therapies include radiation and chemotherapies, surgery, and a combination of any or all of those therapies. The treatments themselves have important side effects, even life-threatening. Chemotherapy is known to impose difficulties because drugs often produce harmful side effects and x-rays sometimes damages normal tissue. Photodynamic therapy offers an alternative, less invasive treatment for such illnesses such as several types of cancers. It involves the use of three basic components [1]: a photosensitizer, a light-absorbing molecule that is activated by the second element, light of a corresponding wavelength, and third, molecular oxygen is consumed during the photochemical reaction to produce cytotoxic agents, thus destroying neoplastic tissue. It is accepted that cell photo-killing (induced in cultured cells) may involve all three main cell death morphologies described, i.e. apoptotic, necrotic and autophagy cell death [2]. Dynamic modeling of cell fate exists, for apoptosis/necrosis [3] and for autophagy [4], in a single cell model. In a previous work [5] we established a model of oxygen transport and cell killing in Type II PDT. This model can be directly linked to these cell fate models, to provide a coherent model of the major biochemical events in PDT on the basis of major components and the main features of the intracellular interactions. Based on existing system biology models [3-5] it is possible to develop a detailed a molecular interaction diagram that summarizes the major biochemical features of the photochemical processes, together with a corresponding system of molecular interactions, rate equations, reaction constants and initial conditions. In the modeling and simulation sections we briefly summarize these major facts of the cell biochemistry of Type II PDT, since the focus of this contribution is the study of the cell decision mechanism of a single cell model in response to PDT treatment and the probability of cell survival.

Information can be defined in terms of its ability to increase the probability of something being true [6] and it is carried on a channel which is a physical mechanism for communication. A channel is distinguished by having a limit on its ability to carry information and by the fact that it is susceptible to random interference, called noise [6]. Whenever energy is transferred, information is transferred. In PDT, light energy is absorbed by the photosensitizers and then transferred to oxygen and other molecules, through a cascade of reactions in the environment of a cell [7]. The PDT treatment parameters act as a "source" generating the input information that the system of molecular network and interactions within a cell must communicate to the "receiver" or the cell [7]. Information is encoded by the parameters of the light and the photosensitizer doses as the source "words" or "code" (death signals) and is transformed into a form, through activated photosensitizers, that can be transmitted through the "channel" of molecular interactions. When decoded by molecular "thresholds", the input information can be converted to a channel output that has the form of a cell's state in terms of necrosis, apoptosis, autophagy or survival [7]. The performance-efficiency of such a bio-communication system and its usefulness for modeling the experimental data is quantified through the assignment of numerical values to the variations and errors that the system may produce. In particular, the mechanism that governs the generation of the source signal and the distortion measure that penalizes the bio-coding errors and determines the fidelity of the reproduction of the cell killing signal need is identified through measurable quantities - functions. Ultimately, the goal is to design and model an optimal treatment strategy that, through the scheme of intracellular biochemical reactions, may lead to reproduction of the PDT death signal output after processing by the cell, with an average distortion that does not exceed a specified upper level D, for a single tumor cell model (or in general, a tumor cell population).

The treatment pattern of the a priori setting parameters (light density, photosensitizer concentration, etc.), is related to the data bio-compression of the death signal through molecular interactions, and the classification of the signal as to cell death or cell survival is done with a possible statistical error that is assigned a numerical penalty: the distortion function or distortion measure *d*[7]. The distortion function *d *does not in itself wholly determine a cell decision. What is important is the relationship between the distortion function and the prior probability distributions of cell death signaling molecules. It is possible to have two different distortion functions which lead to the same decision when the prior probability distributions associated with each, compensate for the details of each distortion function. Combining the three elements of the prior probability (distributions of molecular concentrations), the cell data (distortion tolerance of the cellular system), and the distortion function then allows cell fate decisions to be based on minimizing the mutual information between input and output. The minimization of the mutual information as an application of rate distortion theory to decision making mechanisms in biology has been adapted for testing a framework for designing and analyzing binary decision-making strategies in cellular systems [8], for the information-theoretic characterization of the optimal gradient sensing response of cells [9] and for the rate distortion approach to protein symmetry [10] among other applications. The mutual information of two random variables in general, measures the information that × (input) and Y (output) share: it measures how much knowing one of these variables reduces the entropy of the other. This reduction of the entropy will be compensated by the cell either by interaction with the environment, while it is in a vulnerable state and its survival probability decreases, or by cell death.

There are two kinds of cell division: mitosis and meiosis. Mitosis is essentially a duplication process: It produces two genetically identical "daughter" cells from a single "parent" cell. All cells must replicate their DNA prior to cell division. This assures that each new cell produced receives all of the genetic material necessary to survive and reproduce. Therefore certain information that is formless and does not change or die nor is it composed of matter, is "carried" from one cell to the next and is "reproduced" and it is always present in every cell structure.

John Von Neumann posed and solved the following question: what kind of logical organization is sufficient for an automaton to control itself in such a manner that it reproduces itself? [11]. A cellular automaton is specified by giving a finite list of states for each cell, a distinguished state called the blank state, and a rule which gives the state of a cell at time t+1 as a function of its own state and the states of its neighbors at time t. It consists of a cellular space and a transition function defined over this space. Finite automata constitute the basis of Turing machines [11]. Von Neumann was the first to provide an algorithmic model of a self-reproducing automaton, the Universal Constructor, a self-replicating machine in a cellular automata environment and (in a brief summary) he proved that the construction of this sort of automaton would necessitate the solution to four fundamental problems [11,12]:

α. to store instructions in a memory;

β. to duplicate these instructions;

γ. to implement an automatic factory ("Universal Constructor"), able to read the memory instructions, and, based on them, to construct the components of the system;

δ. to manage all these functions by means of a central control unit.

A self-reproducing system must contain the program of its own construction. This program is a sort of consistent and complete abstract image of the system. In other words, self-reproduction needs programming and processors (software -for information based replication- and hardware). The solution to these problems mentioned above may be found in living things as observed by modern biology. An efficient mechanism of information storage and an elegant mechanism of duplication of the DNA molecule may be the one and only perfect solution to the twin problems of information storage and duplication for self-replicating automata [12]. But more importantly, Von Neumann understood that any information-based replicator must contain inside itself (among other indispensable things) a symbolic representation of itself, an "image" of itself. The relation between the replicator (hardware) and the image (a structure of symbols, the software) is a functional relation of dependence, since the symbolic representation consists of directives and instructions that must be interpreted by the replicator machinery for constructing a copy of itself. As reported by Luis Rocha in his 2012 Fall lecture notes "Biologically-inspired computing", Indiana University, Von Neumann proposed this scheme before the structure of the DNA molecule was uncovered by Watson and Crick, though after the Avery-MacLeod-McCarty experiment which identified DNA has the carrier of genetic information.

With respect to Von Neumann's Universal Constructor we need to notice first that the four principles (α,β,γ,δ) mentioned above are irreducible in complexity and secondly that the concept of the symbolic representation-based self-reproduction implies a language (a symbol system, a syntactic code to be used to map instructions into construction commands for replication. In copying a description, the syntactic aspects are replicated. This indicates that the appropriate framework for the study of such systems could be information theory as discussed below. It is important to decipher the meaning of information available to a cell as something that determines its activity. Information has no mass, energy, or spatial extension, it cannot be seen, touched, or smelled. Nevertheless it is a distinct, objective entity. The cell, as an information system has the ability to discriminate and select between cell fates (which is what we call cell decision making). In fact, the manifestation of information can be found in the existence of alphabets (where as alphabet we interpret the set of physical states that can be realized in some system), the combination of codes (where as a code we consider a collection of the letters of alphabets that follow some pattern-words) and the variety of codes that determine the state of the system.

## Methods

### A brief summary of major molecular pathways and biochemical events induced by PDT

**Figure 1:**
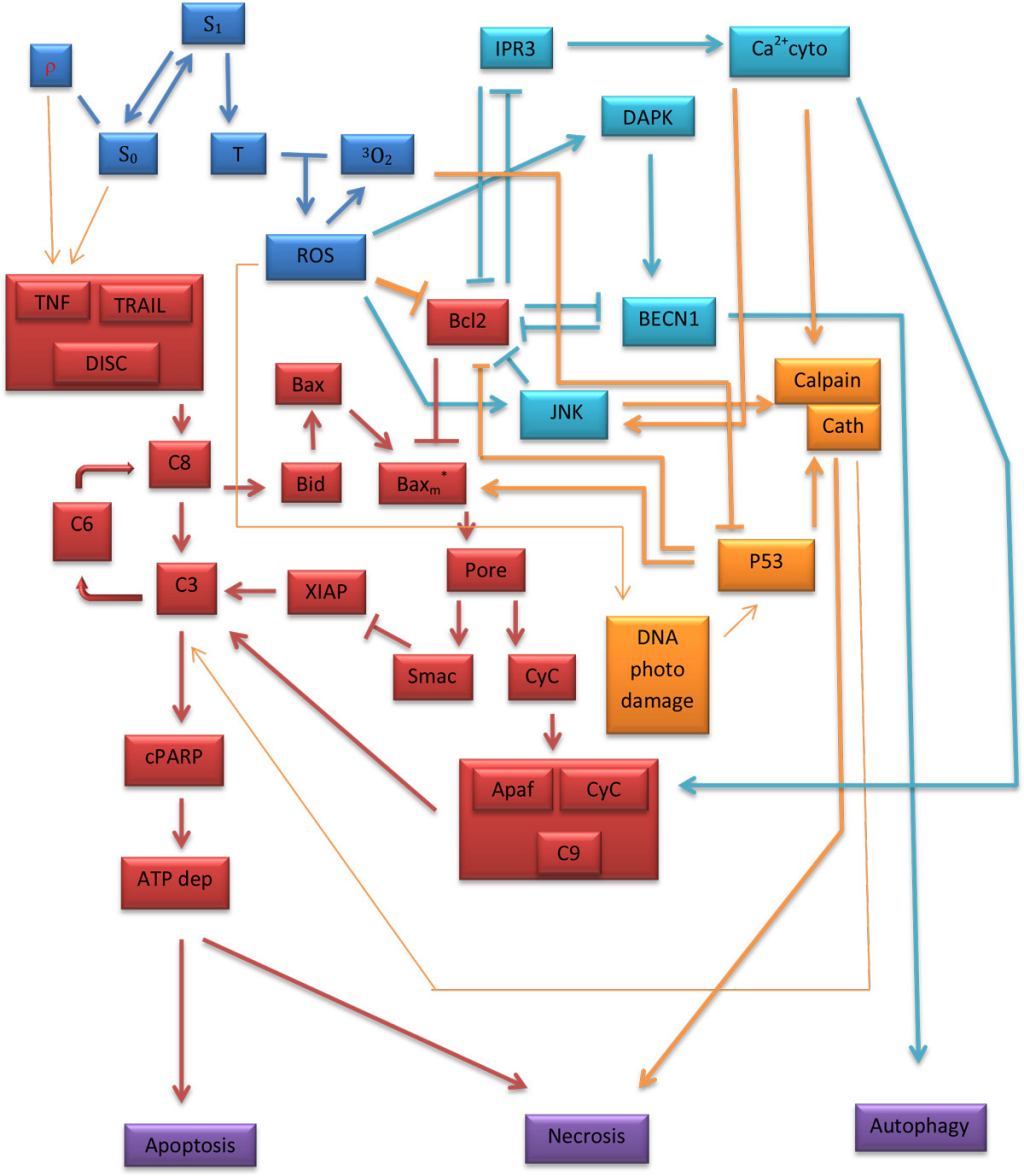
**Schematic representation of the known molecular pathways and biochemical events induced by PDT**. A schematic representation of the known molecular pathways and biochemical events induced by PDT treatment leading to cell death. A molecular interaction graph with information about the potential dynamic behavior of a cellular system that can be translated into mathematical terms that are suitable for computer simulation. Blue color: Reactions that lead to the activation of the photosensitizer(Photofrin in our case) and the generation of reactive oxygen species (ROS). The kinetic mathematical model of [5] is used to describe the molecular pathways. Red color: Exposition to tumour necrosis factor (TNF) or TNF-related apoptosis-inducing ligand (TRAIL) as a result PDT treatment activate effector caspases that dismantle the cell. The kinetic mathematical model of [3] is used to describe the molecular dynamics. Light blue (aqua): The interplay between autophagy and apoptosis in response to oxidative stress. A signaling mathematical model introduced in [4] is used to describe the dynamics. Yellow color: Components and interactions that have either been observed or conjectured in the literature but no equation has been identified (thin lines) or they have been observed and we have a mathematical equation to describe them (thick lines). An arrow signifies the up regulation process of a cellular molecular component as a signalling response to another molecular component, and a line with a vertical line segment signifies the down regulation process of a cellular molecular component as a signalling response to another molecular component. A coherent PDT mathematical model has been synthesized from the three kinetic models, with the necessary modifications to account for biochemical events, such as the initiation of the degradation process of Bcl-2 by ROS that could bind to Bax to prevent its activation. All rate equations, initial molecular concentrations, coefficients and constants can be found in the supplementary information tables in the author's dissertation ("*Mathematical Modelling of Oxygen Transport, Cell Killing and Cell Decision Making in Photodynamic Therapy of Cancer Gkigkitzis, Ioannis ECU, 2012*) as well as the above mentioned references.

We briefly mention here (the biochemistry of PDT has been extensively studied by several other investigators and various publications exist in the medical literature) that in PDT there is a contribution of two reaction mechanisms (Type I and Type II) which result in damage mechanisms that depend on oxygen tension and photosensitizer concentration. Here we adopt our previous PDT modeling scheme of Type II PDT which is considered to be the major reaction mechanism [5]. Singlet oxygen ^1^O_2_, a highly reactive state of oxygen, is a cytotoxic agent generated during PDT treatment. Singlet oxygen is produced during PDT via a triplet-triplet annihilation reaction between ground state molecular oxygen (which is in a triplet state) and the excited triplet state of the photosensitizer (which then returns to its singlet ground state). First, a photosensitizer, a light-absorbing molecule that alone is harmless and has no effect on tissue, is activated by the second element, directed light of a corresponding wavelength that is delivered to the patient [7], selectively targeting the abnormal tissue. Molecular oxygen is consumed during the photochemical reaction to produce cytotoxic agents, thus destroying neoplastic tissue. Besides singlet oxygen and other reactive oxygen species (ROS), activation of caspase cascades known as "executioner caspases" such as caspase-3, -6 and -7 is the next step of the apoptosis/necrosis process [13]. The active executioner caspases cleave cellular substrates, which leads to characteristic biochemical and morphological changes observed in dying cells. Active caspases are potent effector of post-treatment cell apoptosis: For the intrinsic cell death pathway, apoptosis is triggered by intracellular events such as DNA damage and oxidative stress. For the extrinsic cell death pathway, apoptosis is triggered by extracellular stimuli such as TNF and TRAIL. A sharp increase in the levels of caspase 3 indicates the beginning of apoptosis. A very early step upon illumination is cytochrome c release from the mitochondria into the cytosol of treated cells [7]. A possible correlation exists between the cytochrome c release and the loss of the mitochondrial membrane potential and might be related to MOMP (mitochondrial permeability transition pore). Calcium ion release through MOMP is correlated to cytochrome c loss. PDT has a very subtle effect on mitochondrial membrane. Cells could die from ATP depletion (necrosis) or indeed follow the apoptosis activation of the caspase-pathway. Caspase 3 is the caspase that cleaves a large number of proteins that are involved in cell structure and maintenance, such as PARP. Cleaved PARP has been used as the marker of the apoptotic extent. PDT treatment with Pc 4, BPD, or aluminum phthalocyanine (AlPc) has been shown to lead to cleavage of PARP in different cell lines [7,14]. A schematic representation of known molecular pathways and biochemical events induced by PDT is given in (Figure 1).

### Rate distortion theory

**Figure 2:**
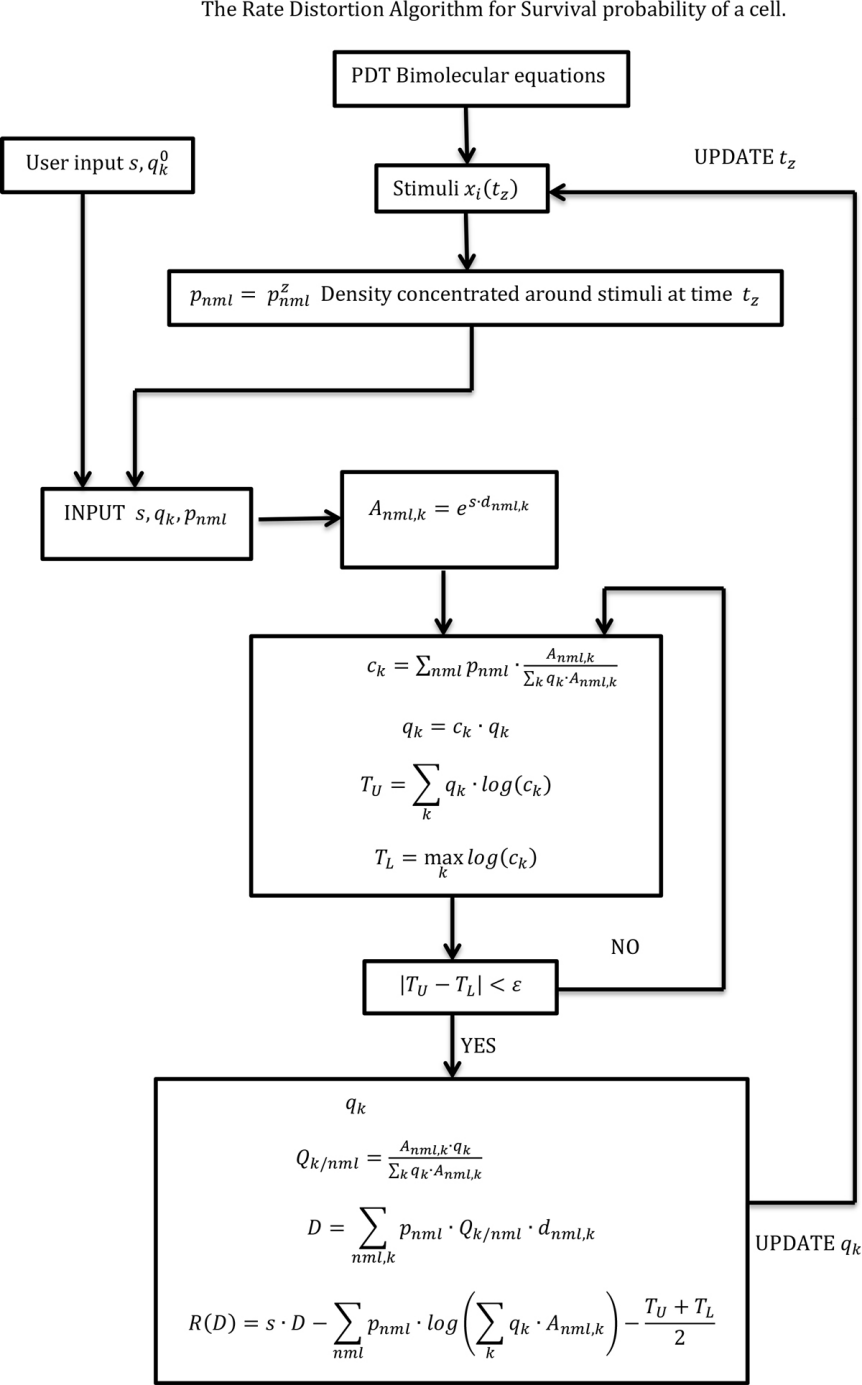
**The Rate Distortion Algorithm for Survival probability of a cell**.

An input vector signal will represent the combined stimulus of different PDT cell death inducing-molecules (Figure 2). We have previously shown [7] in that we can solve an ODE group that describes the major molecular interactions and levels of concentrations during PDT treatment in time domain and obtain the normalized concentrations $X\left(t\right)=\left(x_{1}\left(t\right),x_{2}\left(t\right),x_{3}\left(t\right)\right)$ where:

(1) $$x_{1}(t)={[}^{1}O_{2}]/{{[}^{1}O_{2}]}_{\text{max}},x_{2}(t)=[cPARP]/{\left[cPARP\right]}_{\text{max}},x_{3}(t)=[Casp3]/{\left[Casp3\right]}_{\text{max}}$$

PDT treatment starts at *t *= 0, and ends at a later time *t = t_d _*(this can be 10 to 30 minutes and determines the optical dose for a given photon density). Observation ends at *t = t*_max _= 30 hours. We define $p\left(x_{1},x_{2},x_{3}\right)$ as the probability by which the source produces the "word" (normalized molecular concentration levels) $x\left(t\right)=\left(x_{1}\left(t\right),x_{2}\left(t\right),x_{3}\left(t\right)\right)$[15] at time *t *. For a continuous distribution, starting with *ε *= 0.001 or *ε *< 0.001 we define the bump function approximation to the delta function, in the phase space of the normalized concentrations:

(2) $$p\left(x_{1},x_{2},x_{3}\right)=\frac{1}{\varepsilon^{2}}\eta \left(\frac{x_{1},x_{2},x_{3}}{\varepsilon }\right)$$

(3) $$\eta \left(x_{1},x_{2},x_{3}\right)=\eta \left(x\right)=\left\{\begin{array}{cc} \text{exp}\left(-\frac{1}{1-|x{|}^{2}}\right) & if\;|x|\;<1\\ 0 & if\;|x|\;\geq 1 \end{array}\right)$$

We notice that this is a molifier of the Dirac delta function (*η_ε _*converges to *δ *in the sense of measures, $\eta_{\varepsilon }\overset{L_{1}}{\underset{}{\to }}\delta$), and for values of *ε *small enough, the radius of the compact support is shorter than the step size of the spatial grid of the simulations. We initialize the marginal probability distribution of the cell decision with binary values for the variable y as equal to:

(4) $$q\left(y\right)=\left\{\begin{array}{ccc} q_{0} & for & y=0\left(death\right)\\ 1-q_{0} & if & y=1\left(survival\right) \end{array}\right)$$

A distortion measure is defined as $d\left(x_{1},x_{2},x_{3}|y\right)$, which is a measure of the penalty charged for reproducing the strength of the cell death signal described by the vector stimulus $x=\left(x_{1},x_{2},x_{3}\right)$ by the decision  y and thus quantifies how disadvantageous a given decision  y is in response to a given stimulus  x. The most common distortion measures the Hamming distortion measures of the form:

(5) $$d(x_{1},x_{2},x_{3}|y)=\{\begin{array}{ccc} & x_{i}\geq x_{i}^{th} & x_{1}<x_{1}^{th},x_{2}<x_{2}^{th},x_{3}<x_{3}^{th}\\ y=death & 0 & d_{1}\\ y=survival & d_{2} & 0 \end{array}$$

Where the first inequality hold for at least one $x_{i}$ and *d*_1_, *d*_2 _are real positive numbers. The distortion function describes the goals of a decision-making pathway by quantifying how disadvantageous, or ''distorted,'' a decision  y is in response to a stimulus $x=\left(x_{1},x_{2},x_{3}\right)$. In our case, suppose that when all molecular concentrations are below a threshold $x_{i}^{th}$, the cell should not die; for at least one concentration greater than its fixed threshold $x_{i}^{th}$, the cell should die. In practice, the thresholds may not be clear and a cell can be forgiven for make either decision in response to a stimulus close to the threshold. To represent this situation, a graded distortion function needs to be used [8]. The mechanism by which data is gathered, stored, and utilized by the cell are poorly understood, and rate distortion theory may provide some insight into this function [8]. The "decompressed" data strength (cell decision) may be different from the original data (level of cell death stimulation). Typically, there is some distortion between the original and reproduced signal. This distortion measure, may be cell dependent, or time dependent, describing essential features of a cell, such as how does a cell estimate the state of its environment, how does it quantify alternative decisions, and how does it relate these decisions to the maximization of the fitness of the population [8]. In other words, the distortion measure is the penalty for an incorrect classification of the level of a molecular concentration, which leads to errors in the stimulus pattern recognition, which in this frame is the assignment of a cell fate probability to the given input by the bio molecular reactions. For the purposes of information theory a discrete channel is described by a probability transition matrix $Q\left(y|x_{1},\;x_{2},\;x_{3}\right)$ where *Q *is the conditional probability of receiving the  y output-signal letter given that the $\left(x_{1},\;x_{2},\;x_{3}\right)$ input letter signals were transmitted. As a conditional probability it is related to the probability distributions of the random vector $x=\left(x_{1},x_{2},x_{3}\right)$ and the random variable  y, by the equation:

(6) $$q\left(y\right)=\underset{x_{1}x_{2}x_{3}}{\sum }p_{X}\left(x_{1},x_{2},x_{3}\right)Q\left(y|x_{1},x_{2},x_{3}\right)$$

**Figure 3:**
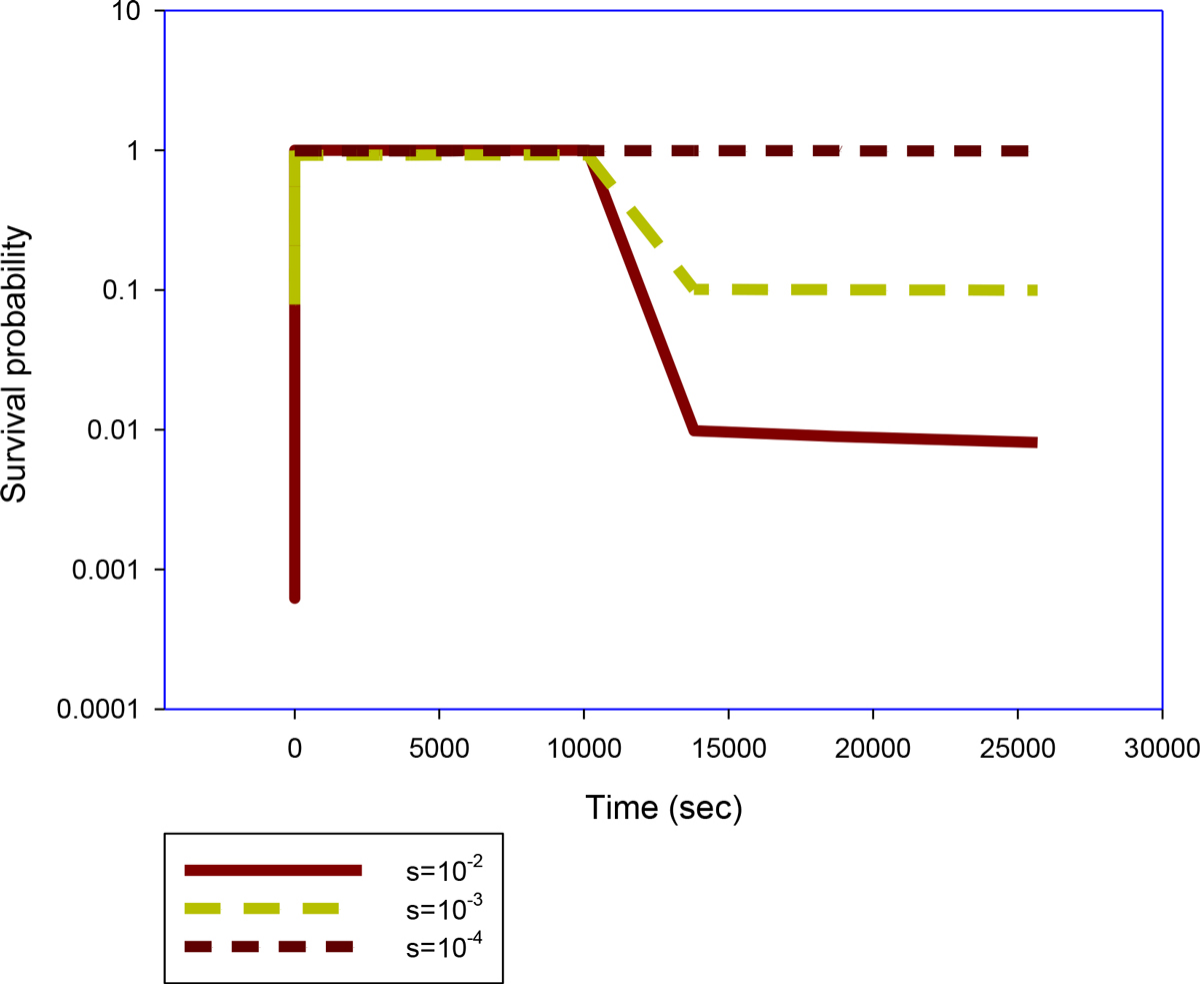
**Survival probability curves**. A sample of a survival probability curves as predicted by the Blahut Arimoto algorithm for the cell model. Value of the parameter *s *= -10^-2^, -10^-3^, -10^-4 ^Photon density *ρ *= 10^6 ^*cm*^-3^. Photo sensitizer (Photofrin) concentration in a cell [*S*_0_] = 5 × 10^13 ^*cm*^*-*3^. Single cell oxygen concentration [^3^*O*_2_] = 6.06 × 10^17 ^*cm*^-3^.

In the minimization of the mutual information defined below, the conditional probability matrix Q will be calculated through a relation that is derived by the method of Lagrange multipliers [8,15]:

(7) $$Q\left(y|x_{1},x_{2},x_{3}\right)=\frac{q\left(y\right)\cdot e^{s\cdot d\left(x_{1},x_{2},x_{3}|y\right)}}{\underset{y^{\prime }}{\sum }q\left(y^{\prime }\right)\cdot e^{s\cdot d\left(x_{1},x_{2},x_{3}|y^{\prime }\right)}}$$

where s is taken to be a negative number. This is the Lagrange multiplier for the method of calculus of variations, which is used to find the optimal cell decision probability q and conditional probability *Q *by minimizing the average mutual information between source (stimulus vector) and receiver (cell/cell decision) [16] (Finding extrema of a function is a most common problem, but difficulties often arise when one wishes to maximize or minimize a function subject to fixed outside conditions or constraints. The method of Lagrange multipliers is a powerful tool for solving this class of problems without the need to explicitly solve the conditions and use them to eliminate extra variables). We calculate the strategy as defined by equation (5) and (6) that minimizes the average mutual information between the input $\left(x_{1},\;x_{2},\;x_{3}\right)$ (death stimuli, normalized concentrations) and the output (decision  y), and the decision probability for cell survival (Figure 3) or cell death by implementing a time dependent optimization Blahut Arimoto algorithm (Figure 2). The average mutual information is defined as [15,16]:

(8) $$I\left(p_{X},Q\right)=\underset{x_{1},x_{2},x_{3},y}{\sum }p_{X}\left(x_{1},x_{2},x_{3}\right)Q\left(y|x_{1},x_{2},x_{3}\right)\text{log}\frac{Q\left(y|x_{1},x_{2},x_{3}\right)}{q\left(y\right)}$$

The rate distortion function *R*(*D*) is defined as

(9) $$R\left(D\right)={\text{min}}_{Q\in Q_{D}}I\left(p,Q\right)$$

where, *Q_D _*is defined as the collection of all conditional probabilities-strategies $Q\left(y|x_{1},x_{2},x_{3}\right)$ such that *d*(*Q*) ≤ *D *where the expected distortion is given by [16]

(10) $$D=\underset{x_{1},x_{2},x_{3},y}{\sum }p_{X}\left(x_{1},x_{2},x_{3}\right)Q\left(y|x_{1},x_{2},x_{3}\right)d\left(x_{1},x_{2},x_{3}|y\right)$$

The function *R*(*D*) describes the amount of information needed to be preserved by this biochemical data compression scheme of the source output which is given in the form of levels of molecular concentrations, so that reproduction of the death/survival signal can be subsequently generated from the compressed data with average distortion less than or equal to some specified value *D *(the rate distortion function *R*(*D*) has been initially defined as the effective rate at which the source produces information and passes it to the "user", subject to the constraint that the "user" can tolerate an average distortion D [16]). According to [8] the complexity and (metabolic) cost of a channel generally varies directly with its capacity, and a less complex strategy is more likely to be followed and be realized by a biological system. With minimal information *I *and a cell dependent distortion measure *d*, the optimal strategy incorporates randomness to generate biological variation. With respect to the distortion measure *d*, following [8] one can notice that while this analysis requires knowing the probability distribution of stimuli as well as the conditional probability distributions of probabilities-strategies, an experimental estimate of these distributions through survival curves can produce an approximation of the distortion function around which the pathway is optimized. Starting from:

(11) $$Q\left(y=y_{2}|x_{1},x_{2},x_{3}\right)=\frac{q\left(y_{2}\right)\cdot e^{-s\cdot \left(x_{1},x_{2},x_{3}|y\right)}}{q\left(y=y_{1}\right)\cdot e^{-s\cdot d\left(x_{1},x_{2},x_{3}|y=y_{1}\right)}+q\left(y=y_{2}\right)\cdot e^{-s\cdot d\left(x_{1},x_{2},x_{3}|y=y_{2}\right)}}$$

And solving for the distortion measure, we get:

(12) $$d\left(x_{1},x_{2},x_{3}|y=y_{2}\right)=\frac{1}{s}\cdot \text{log}\left[\frac{Q\left(y=y_{1}|x_{1},x_{2},x_{3}\right)\cdot q\left(y=y_{2}\right)}{Q\left(y=y_{2}|x_{1},x_{2},x_{3}\right)\cdot q\left(y=y_{1}\right)}\right]+d\left(x_{1},x_{2},x_{3}|y=y_{1}\right)$$

We can pick $d\left(x_{1},\;x_{2},\;x_{3}|y=y_{1}\right)$ such that

(13) $$\text{min}\left\{d\left(x_{\text{1}},x_{\text{2}},x_{\text{3}}|y=y_{\text{2}}\right),d\left(x_{\text{1}},x_{\text{2}},x_{\text{3}}|y=y_{\text{1}}\right)\right\}=0$$

And this determines the distortion measure.

### Logical design

Computation of cell decision discrete probability q(y=survival), distortion *D *and rate distortion function *R*(*D*) is performed using the method of Lagrange multipliers. The problem is solved computationally using the Blahut-Arimoto algorithm. We summarize the basic steps:

a) Assign a value ε that determines the accuracy of the algorithm.

b) Initialize the exponential function $e^{s\cdot d\left(x_{1},x_{2},x_{3}|y\right)}$.

c) Initialize the probability distribution of the decision $q^{0}\left(y\right)=q\left(y\right)$ as a binary distribution as in equation (4).

d) Given q(y) compute the conditional probability distribution $Q\left(y|x_{1},\;x_{2},\;x_{3}\right)$ that minimizes the mutual information $I\left(p_{X},\;Q\right)$ while satisfying the condition of equation (6).

e) Update the probability q(y) that minimizes the mutual information by using equation (5).

f) Calculate the distortion D given by the equation (9).

g) Calculate the rate distortion function given by [16]:

(14) $$R(D)=s\cdot D-\sum_{x_{1},x_{2},x_{3}}p_{X}(x_{1},x_{2},x_{3})\cdot \log\left(\sum_{y}q(y)\cdot e^{s\cdot d(x_{1},x_{2},x_{3}|y)}\right)-\frac{T_{U}+T_{L}}{2}$$

The convergence of the algorithm depends on the difference between two bounds the lower and upper bound ([15],Theorem 7):

(15) $$T_{L}={\text{max}}_{y}\text{log}\left(c\left(y\right)\right)$$

(16) $$T_{U}=\underset{y}{\sum }p\left(y\right)\text{log}\left(c\left(y\right)\right)$$

The algorithm gives better convergence for small difference between these two bounds in absolute value:

(17) $$|T_{U}-T_{L}|\;<\varepsilon$$

h) Iterate steps (a-g) for all simulation times *t*, and this yields $q^{t}\left(y=survival\right)=q_{survival}\left(t\right)$ for one set values of PDT treatment parameters: photo-density *ρ*, drug concentration [*S*_0_] and initial molecular oxygen concentration ${\left[{}^{3}O_{2}\right]}_{i}$.

i) (optional) Vary (${\left[{}^{3}O_{2}\right]}_{i}$, [*S*_0_], [*F*]) where *F *is the fluence, ${\left[{}^{3}O_{2}\right]}_{i}$ is the molecular oxygen concentration in the cell, [*S*_0_] is the concentration of ground state photosensitizer, and ([*F*]is the fluence (see [5]) and obtain corresponding survival curve $q_{survival}=q({[}^{3}O_{2}]i,[S_{0}],[F],t)$ for optimization of fluence/drug dose modeling parameters for the biomolecular mechanism studied and the given choice of molecular components of the stimulus vector). Fit to experimental data of survival curves to find the optimal range for the parameters.

### Survival functions - predator prey models

Survival functions can be derived using predator-prey model. The predator-prey model has been used for the description of the survival probability in dynamic energy budget models [17] under the assumption that that the per capita death rate has two contributions, a constant loss due to random misfortunes, and a density-dependent loss due to predation, with a Holling Type II functional form. This model was designed to predict the growth and reproduction patterns of a species based on the characteristics of individual organisms, particularly the strategy used to allocate resources.. This model takes an individual-based approach where all members of the prey population are "copies" of one individual, and each "copy", could be the "model individual" itself. The use of a predator-prey model (a continuous model used for the simulation of discrete population dynamics) for the modeling of survival probability(a continuous variable) suggests the quantization of survival probability. Indeed, the quantization of probability has been proposed by other authors [18,19]. The existence of the "chance-quantum" (c.q.), implies certain axioms [Go 43]. For example, if the probability of an event is equal to or greater than one c.q., it may ultimately occur, if an event has a calculated probability of less than one cq. it will not occur, for an event having an appreciable probability (equivalent to many cq.), a change in surrounding conditions leading to a computed change in probability of less than one cq. will in fact cause no change in the probability of the event, etc.

Survival Units Duality refers to the idea that the life a cell (or survival probability) can be discretized (quantized) in quanta of life (survival units) which are assumed here as the basic units of life in every cell. New cells are produced by existing cells, and therefore the termination of a cell does not allow to assign any morphological or biochemical characteristics to the life of the cell itself, since these characteristics can only be considered as the manifestations of the monitoring, interaction and response of the cell, as a biochemical unit undividedly united to cellular life ("life units"), to the extracellular environment. Cellular life (survival probaility) is a set of life units (survival probaility quanta), where each cellular life unit contains the whole complete life of the cell in itself, therefore allowing the cell to repair itself after any loss of survival units due to the attack of cell death inducers or other factors (Figure 3). Therefore, the survival probability of a cell is a set that contains itself within each of its elements (survival probablity quanta or units). This idea is not new in mathematics. This is in accordance with Von Neumann's idea that any information-based replicator must contain inside itself a symbolic representation of itself, an "image" of itself.

### Russell's paradox and information

A set is a collection of objects, or elements. Sets are defined by the unique properties of their elements and sets and elements may not be mentioned simultaneously, since sets are determined by their elements and therefore one notion has no meaning without other. Bertrand Russell, while working on his "*Principia Mathematica*" (Principles of Mathematics) in 1903, he discovered a paradox that arised from Frege's set theory that leads to a contradiction [20]. It says "the set of all sets which are not members of themselves contains itself." In mathematical terms, let S={x:x∉x}, then *S *∈ *S *⇔ *S *∉ *S*. Although the precise rules for set formation have been under intense investigations and several different logical systems have been proposed, sets that contain themselves as elements, like S, are definitely ruled out, as "abnormal". Based on the work Russell and Whitehead, Kurt Gödel was able to show that a theorem could be stated within the context of Russell and Whitehead's system that was impossible to prove within that system [21]. Gödel's Incompleteness Theorem states that there are mathematical statements that can never be proved, in any consistent system of axioms such as the arithmetic system.

The need for the distinction between two kinds of collection can be found back in the work of Schroder and Cantor [22]:

*"If we start from the notion of a definite multiplicity of things, it is necessary, as I discovered, to distinguish two kinds of multiplicities (by this I always mean definite multiplicities). For a multiplicity can be such that the assumption that all of its elements "are together" leads to a contradiction, so that it is impossible to conceive of the multiplicity as a unity, as "one finished thing". Such multiplicites I call absolutely infinite or inconsistent multiplicities.... If on the other hand the totality of the elements of a multiplicity can be thought of without contradiction as "being together", so that they can be gathered together into "one thing", I call it a consistent multiplicity or a "set"*.

Cantor's conclusions are the ancestors of today's distinction between classes and sets, as they appear in the work of Von Neumann [23]. For von Neumann all sets are classes, but not all classes are sets. And those classes that are not sets - the so-called proper classes -cannot themselves be members [22]. In Von Neumann's axiomatization theory, some major advantages are [22]: There are extensions for the predicates 'set', 'non-self-membered set', 'well-founded set', 'ordinal'. There is a well-determined collection of all the Zermelo-Fraenkel sets; and there is a domain for quantification over sets. Further, the Axiom of Choice is provable in von Neumann's system. Several issues, both technical and intuitive, have been reported with respect to this system. A discussion can be found in [22], and here we only mention the consequence of this theory, that the concept of class has no extension (based on the axioms of this system, there is no class of all classes, and therefore the problem has just been pushed back). Therefore the resolution of this paradox remains unresolved.

In mathematical logic, it is suggested that problems that are essentially the same must be resolved by the same means, and similar paradoxes should be resolved by similar means. This is the principle of uniform solution [281]. Two paradoxes can be thought to be of the same kind when (at a suitable level of abstraction) they share a similar internal structure, or because of external considerations such as the relationships of the paradoxes [281]. The question rises as to the existence of other paradoxes that are of the same kind with Russell's paradox. Russell focused more on the underlying structure of the paradoxes and saw them all as paradoxes of impredicativity. The "inclosure schema" was proposed by Priest, as a formal schema that can be used to classify paradoxes [24]. Although the schema will not be analyzed in this work, the conclusion is very interesting: Russell's paradox is of one kind with the "sorites" paradox (the paradox of the "heap"). This paradox was introduced by to Eubulides of Miletus (4th century BC), a pupil of Euclid, and appears when one considers a heap of sand, from which grains are removed. Is it still a heap when only one grain remains? If not, when did it change from a heap to a non-heap? These two paradoxes are neighboring paradoxes, and it has been suggested that we should not just consider the internal structure of the paradoxes, although that is undoubtedly important, but we also consider the external relationships--the relationships to other nearby paradoxes [281]. The way nearby neighbors (paradoxes of one kind) respond or fail to respond to proposed treatments tells us something about what makes the whole family tick and about their structural similarity [281]. The question "when is the cell dead?" indicates confusion between cessation of organic coherence and cellular activity. When a cell irrevocably loses its organization, it's dead. The point when it becomes irrevocably damaged is related to the sorites problem.

It is known that continuous time Markov processes, are used for the formulation of stochastic predator prey models that are based on within individual variation [25]. Within individual variation, used under the name of "demographic stochasticity", has been used in the theory of adaptive dynamics. The theory of adaptive dynamics aim at describing the dynamics of the dominant trait in a population, that is called the 'fittest' trait. The main approach is through stochastic or individual centered models which in the limit of large population, can be transformed into integro-differential equations or partial differential equations [26]. Stochastic simulations, using a finite size population, involve extinction phenomenon operating through demographic stochasticity which acts drastically on small populations [26]. These simulations involve a unit for minimal survival population size, which corresponds to a single individual. In general though, typical stochastic and deterministic simulations do not fit and give rather different behaviors in terms of branching patterns. It has been observed that the notion of demographic stochasticity does not occur in general in deterministic population models, and an alternative proposed has been proposed in order to include a similar notion in these models: the notion of a survival threshold [27], which allows some phenotypical traits of the population to vanish when represented by too few individuals. In particular, through the investigations of simple and standard Lotka Volterra systems that describe the time of the distribution of phenotypic traits in time, it is shown that the inadequacy of deterministic models to handle extinction phenomena through demographic stochasticity, can be corrected by the introduction of a survival threshold, leading to a mimicking effect of the extinction probability due to demographic stochastcity in small sub-populations, while hardly influences the dynamics of large sub-populations [26]. In this framework, the above principle implies (at the extreme) that densities correspond to less than one individual are undesirable [26], indicating that the link between the continuous (large populations) and the discrete (small sub populations), between the existence (survival) and the vanishing (extinction - demographic stochasticity) is correlated with the existence of a survival threshold in the model.

Furthermore, this hybrid approach of survival, as continuous-discrete function with a survival threshold assigned to a population, raises the following question: Is there an internal quantization scheme that relates the continuous models for large populations with survival thresholds to small populations' discrete models? The existence of both features, of continuity and quantization in a single process, appears in the study of the conditional survival probabilities of a firm (the computation of the conditional survival probability of the firm from an investor's point of view, i.e., given the "investor information"). Callegaro and Sagna used a quantization procedure, to analyze and compare the spread curves under complete and partial information in new and more general settings in their work on applications to credit risk of optimal quantization methods for nonlinear filtering. The theory of quantization probability they used was based on an earlier study of local quantization behavior of absolutely continuous probabilities [28]. This study analyzes the *L^r ^*quantization error estimates for *L^r^*(*P*) codebooks for absolutely continuous probabilities *P *and and Voronoi partitions satisfying specific conditions. But the origins of the theory developed there can be traced back to electrical engineering and image processing and in particular in digitizing analog signals and compressing digital images [29]. Therefore, in the heart of the study of survival probabilities we find a theory for the quantization as analog-to-digital conversion and as data compression. Analog signal is a continuous signal which transmits information as a response to changes in physical phenomenon and uses continuous range of values to represent information, where digital signals are discrete time signals generated by digital modulation and use discrete or discontinuous values to represent information. The quality of a quantizer can be measured by the goodness of the resulting reproduction of a signal in comparison to the original. This is accomplished with the definition of a distortion measure that quantifies cost or distortion resulting from reproducing the signal, and the consideration of the average distortion as a measure of the quality of a system, with smaller average distortion meaning higher quality [29].

This is precisely the framework we adopt in this work to study and analyze the process of cell survival during treatment (in our framework). This suggests an organic connection among an axiomatic system foundation, a predator prey rate equation and information theoretic signal processing.

### Mathematical modeling scheme

Following [3], biochemical reaction equations were derived from the following scheme, for the apoptosis/necrosis pathways. For a given binary reaction *i *the biochemical equation is represented by one of following general mass-action paradigms:

(18) $$A+B\underset{k_{-i}}{\overset{k_{i}}{↔}}A:B\overset{\kappa_{i}}{\underset{}{\to }}A+P\Leftrightarrow \left\{\begin{array}{c} \frac{d\left[A\right]}{dt}=-k_{i}\left[A\right]\left[B\right]+k_{-i}\left[A:B\right]+\kappa_{i}\left[A:B\right]\\ \frac{d\left[S\right]}{dt}=-k_{i}\left[A\right]\left[B\right]+k_{-i}\left[A:B\right]\\ \frac{d\left[A:S\right]}{dt}=k_{i}\left[A\right]\left[B\right]-k_{-i}\left[A:B\right]\\ \frac{d\left[P\right]}{dt}=k_{i}\left[A:B\right] \end{array}\right)$$

Where *k_i_, k*_-*i*_ are the reaction rates. For the autophagy pathway, we follow [4] and the neural network modeling method as it is described in [4,30]. We use an intermediate modeling strategy that employs nonlinear ODE to describe protein regulatory networks but is not tied to specific reaction mechanisms and rate constants. More precisely, we use ODEs of the form:

(19) $$\frac{dX_{i}}{dt}=\gamma_{i}\cdot \left[F\left(\sigma W_{i}\right)-X_{i}\right]$$

(20) $$W_{i}=\omega_{i0}+\overset{N}{\underset{j=1}{\sum }}\omega_{ij}X_{j}$$

Where $X_{i}$ is the expression level of a molecular concentration $0\leq X_{i}\leq 1$ and $F\left(\sigma W_{i}\right)=\frac{1}{1+e^{-\sigma W}}$ is a sigmoidal function that varies from 0 (when $W<<-\frac{1}{\sigma }$) to 1 (when $W>>-\frac{1}{\sigma }$). The parameter σ controls the steepness of the sigmoidal function at its inflection point. *W_i _*is the net effect on molecule *i *of all molecules in the network. The coefficient *ω_ij _*is less than 0 if molecule *j *inhibits the expression of molecule *i*, more than 0 if molecule *j *activates molecule *i*, or equal to 0 if there is no effect of molecule *j *on molecule *i*. This equation has the great advantage that it is subject to all the powerful analytical and simulation tools of nonlinear ODEs, yet, in the limit of large *σ*, it behaves like a discrete Boolean network [30]. When *σ *≫ 1, $X_{i}$ tends to flip (on a timescale ≈ *γ*^-1^) between 0 and 1, and the dynamical system approximates a Boolean network [30].

### Modeling and simulation

**Figure 4:**
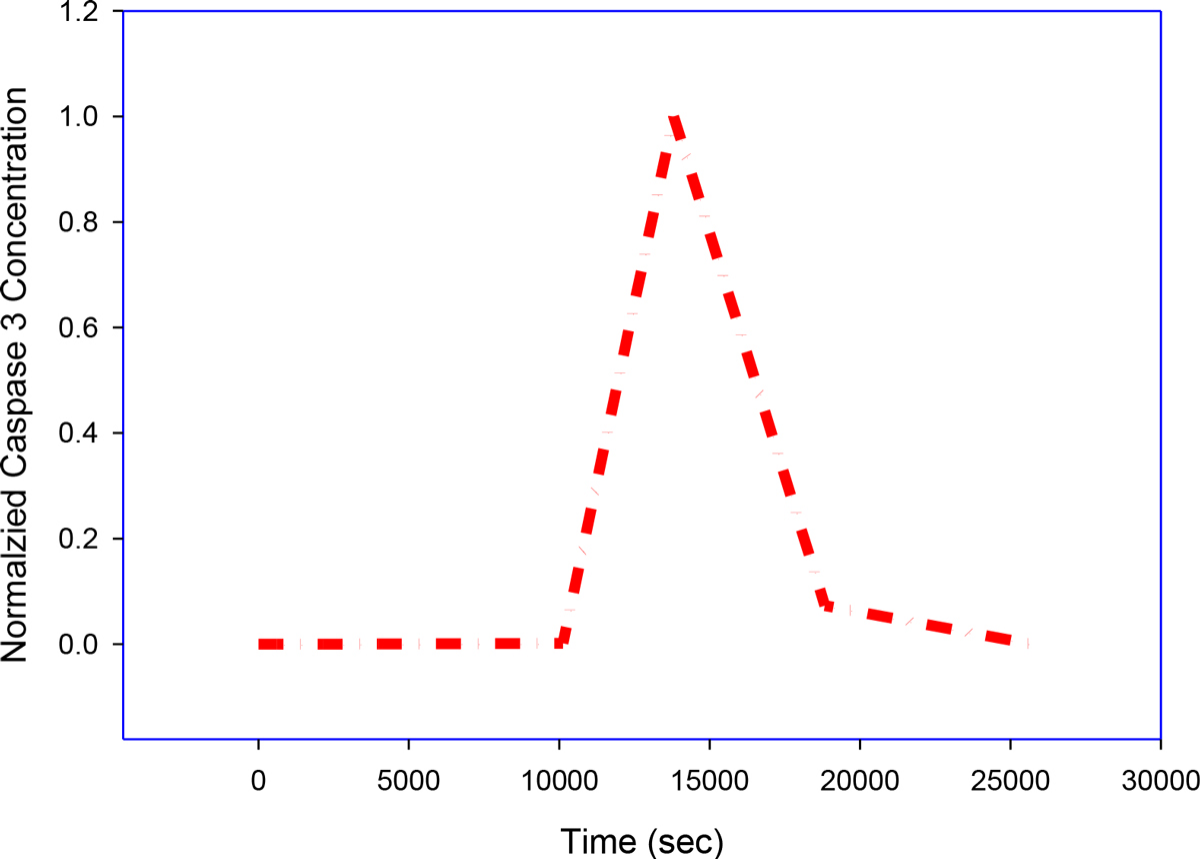
**PARP activation**. PARP activation is an immediate cellular response to metabolic, chemical, or radiation-induced DNA SSB damage. Upon DNA cleavage by enzymes involved in cell death (such as caspases), PARP can deplete the ATP of a cell in an attempt to repair the damaged DNA. ATP depletion in a cell leads to lysis and cell death. PARP also has the ability to directly induce apoptosis, via the production of PAR, which stimulates mitochondria to release AIF. This mechanism appears to be caspase-independent.

**Figure 5:**
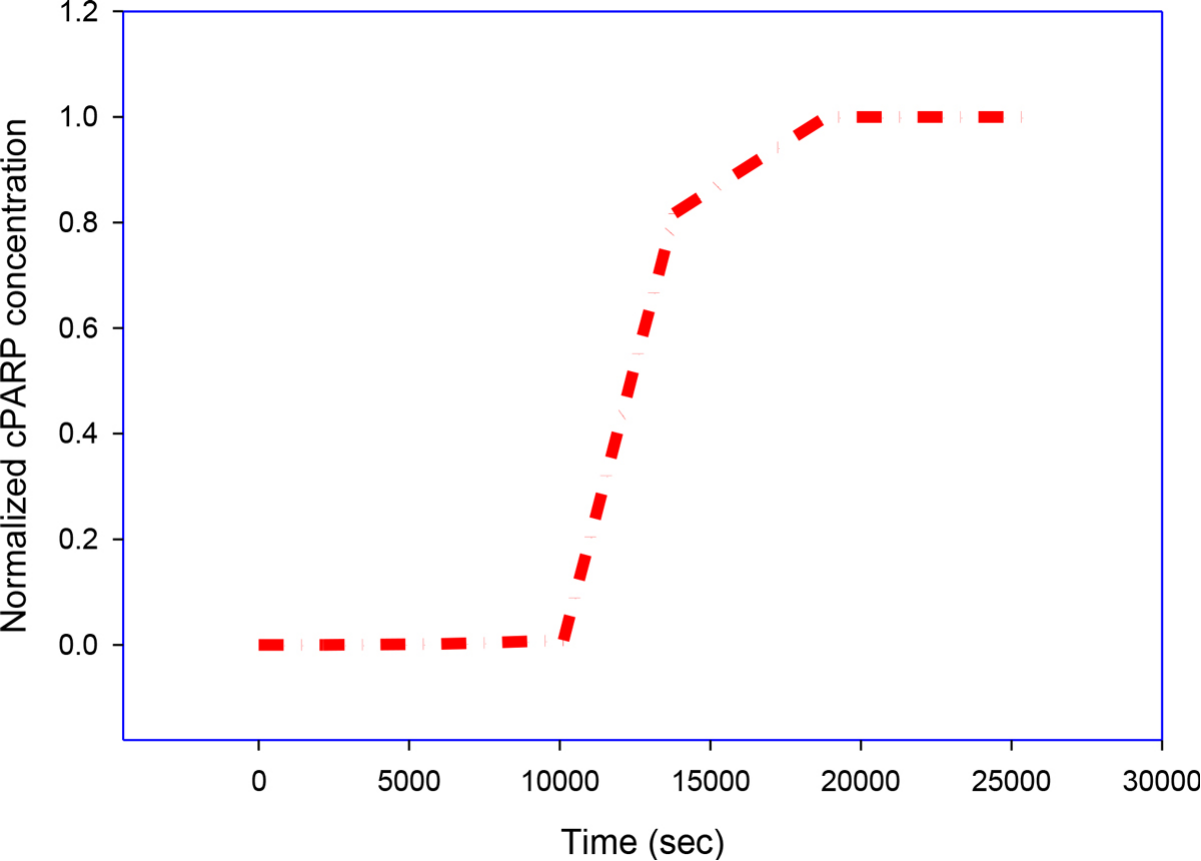
**Singlet Oxygen**. Singlet Oxygen is the most important cytotoxic agent generated during PDT(it decays after photo irradiation time). Singlet oxygen is produced during PDT via a triplet-triplet annihilation reaction between ground state molecular oxygen (which is in a triplet state) and the excited triplet state of the photosensitizer.

**Figure 6:**
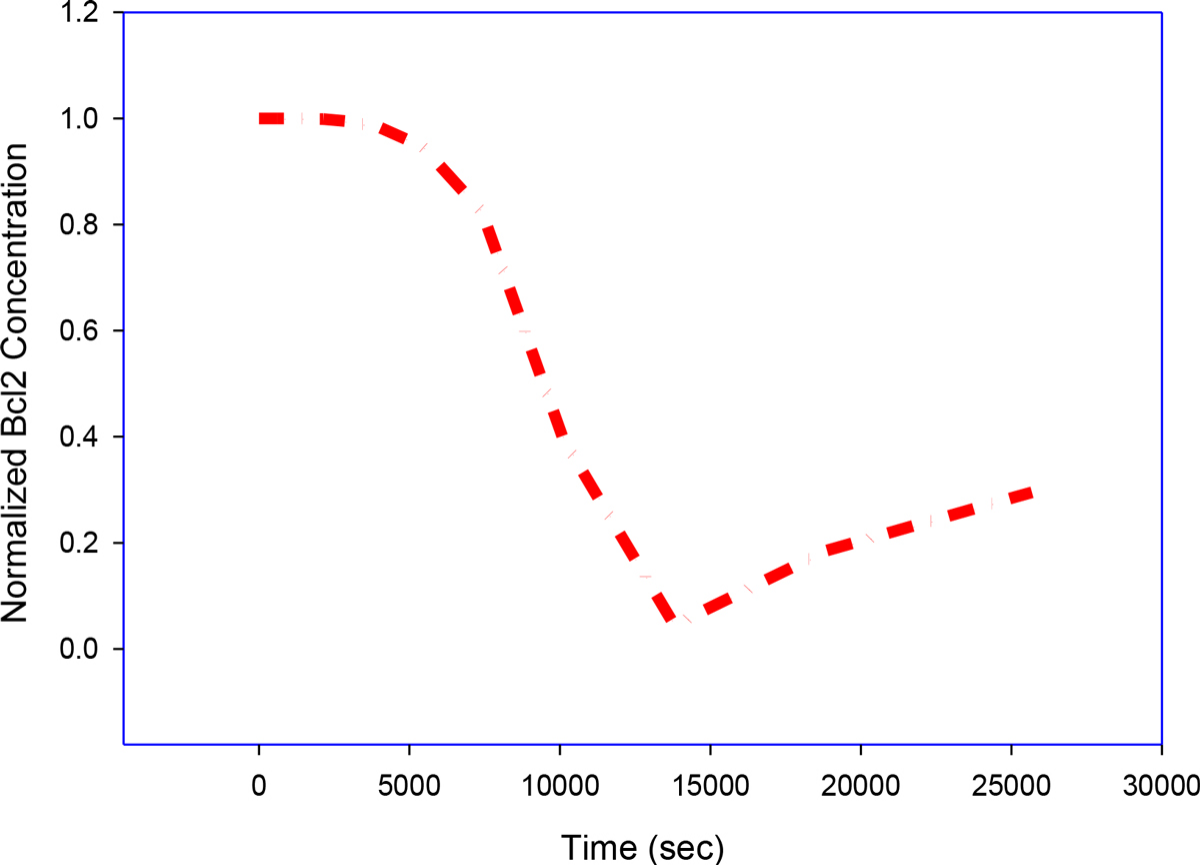
**Bcl-2 degradation**. ROS initiates the degradation process of Bcl-2 that could bind to Bax to prevent its activation. At a later time after the photo-irradiation a post-treatment increase of Bax is observed.

**Figure 7:**
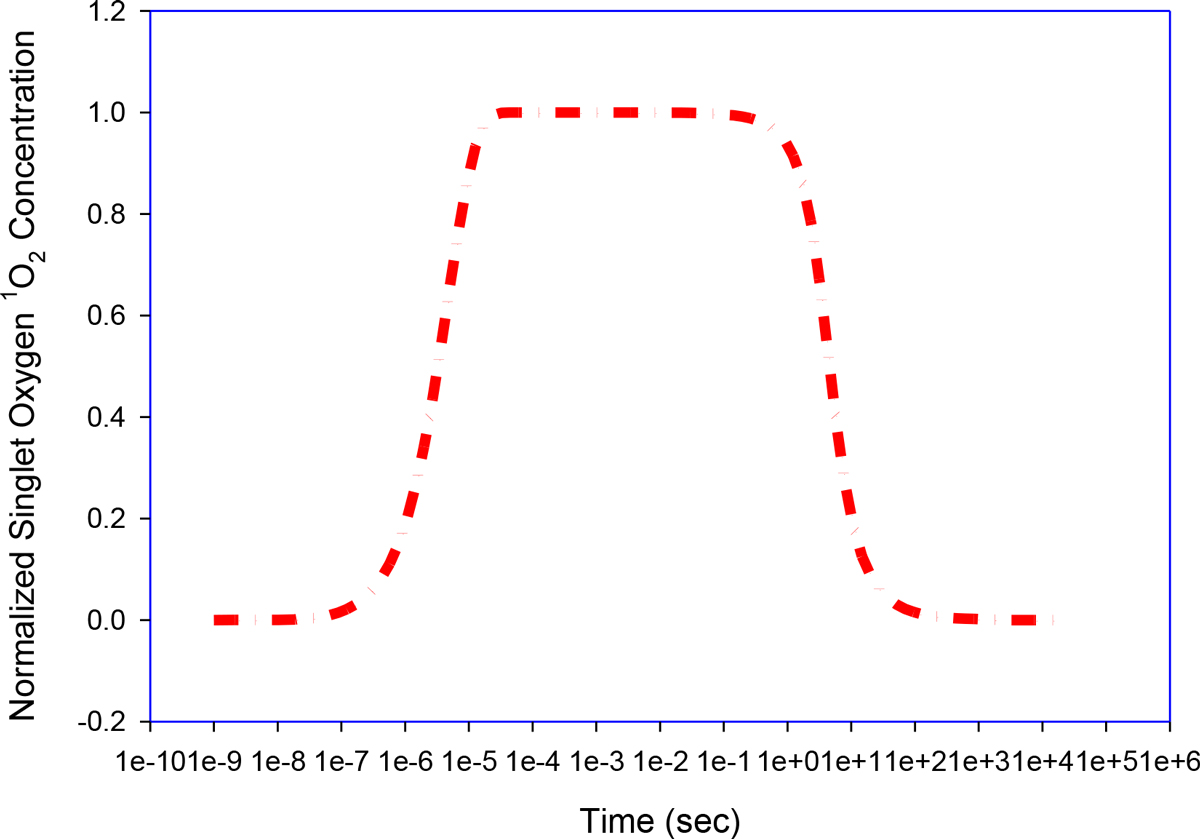
**Caspase 3**. Activated Caspase 3 is potent effector of post-treatment cell apoptosis: For the intrinsic cell death pathway, apoptosis is triggered by intracellular events such as DNA damage and oxidative stress. For the extrinsic cell death pathway, apoptosis is triggered by extracellular stimuli such as TNF and TRAIL. A sharp increase in the levels of Caspase 3 indicates the beginning of apoptosis.

A group of rate equations based on a molecular diagram (Figure 1) can be used to quantitate the time evolution of the following molecule species in a Type II PDT process: photosensitizers (Photofrin) in ground state *S_0_*, single and triple excited states *S_1 _*and *T*; oxygen molecules in triplet grounded and single excited states *^3^O_2 _*and *^1^O_2_*. Death ligand such as *TRAIL *and *TNF*; inactive receptor complex R*;FLICE-like inhibitory protein *flip*; procaspase-8 and procaspase-10, inactive, both as *C8*, bi-functional apoptosis regulator *Bar*; (cleaved) active caspase-8 and caspase-10 C8* ; procaspase-3 and procaspase-7, inactive, both as *C3 *; procaspase-6, inactive capsase-6 *C6*; (cleaved) active caspase-3 and caspase-7 C3* (Figure 4) and active caspase-6 C6*; × linked inhibitor of Apoptosis in the cell *XIAP*; Poly (ADP-ribose) polymerase *PARP* (Figure 5), as DNA damage repair enzyme , here all substrate of active caspase-3 C3*;The BH3 interacting-domain death agonist *Bid *as a substrate of cleaved caspase-8 in its inactive form; the anti-apoptotic protein *Bcl-2* (Figure 6); the Bcl-2-associated × protein in its inactive form *Bax *and its active form Bax* ; *Bax *in the mitochondrial compartment as *Baxm*; cytochrome c inside the mitochondria in the mitochondrial compartment *CyCm *and cytochrome c release from the mitochondria but remaining in mitochondrial compartment *CyCr*; cytochrome c in cellular compartment *CyC *; second mitochondria-derived activator of caspases. *Smac *and *Smac/Diablo *released from the mitochondria but remaining in mitochondrial compartment, *Smacr *; Apoptosis activating factor *Apaf-1*, substrate of *CyC*, in its inactive form Apaf1; active form of Apaf-1, Apaf*; inactive form of procaspase- 9 *C9*; the apoptosome *Apop *which is the complex Apaf*:C9; inositol-requiring protein 1 *IRE1*; JUN N-terminal kinase, *JNK*; death associated protein kinase *DAPK*; Beclin mediator of autophagy phosphorylated by death associated protein kinase *DAPK, BECN1*;the tumor suppressor protein *p53*; the intracellular concentration of calcium *Ca2+*; the protease Cathepsin *Cath *and the protease *Calpain*; the inositol 1,4,5-trisphosphate receptor *IP3R *or *IPR3*; Even though the singlet excited oxygen molecules *^1^O_2 _*may play a critical role by themselves, it is well known that other *ROS *species are also involved in the cytotoxicity of PDT with mitochondria as the possible source and target sites. The *1O2 *should be interpreted as the representatives of *ROS *(Figure 7). The molecular equations, together with the definitions of coefficients and their values that were used to mathematically model the molecular network described above have been detailed in [3-5] and have presented by the author in his dissertation thesis (see below legend of Figure 1). A 70 equation group can be solved to characterize the main molecular interaction involved in Type-II PDT. We use the ordinary differential equation (ODE) stiff solver (ode15s) by MATLAB (The MathWorks, Natick, MA) to obtain the solution vector as a function of illumination time t from the start of illumination at t = 0 to 1800 (s). Experimental verification of these quantities that describe the levels of all these molecular concentrations can be very difficult if not impossible and they relate indirectly to the ultimate consequence of PDT for cell killing. In [5] we introduced a cell killing model that related the molecular concentrations of the singlet oxygen and the unoxidized receptors to the cell survival ratio, which can be measured with an in vitro cell model. A system of 70 ODE (ordinary differential equations) was solved numerically to characterize the main molecular interactions involved in Type-II PDT. The output of this equation group is the time dependent levels of molecular concentrations for the stimulus vector of $x\left(t\right)=\left(x_{1}\left(t\right),x_{2}\left(t\right),x_{3}\left(t\right)\right)$ corresponding to singlet oxygen *^1^O_2_, cPARP *and *Caspase 3*. The concentrations were normalized with respect to their maximum values and their range is 0[1].

The total time for the simulations was up to 30,000 sec to monitor post-treatment cell killing. We used the stiff solver (ode15s) by MATLAB (The Math Works, Natick, MA) to obtain the solution vector as a function of illumination and observation times, from the start of illumination at t = 0 to 1800 (s) (end of illumination time) and from 1800 to 30,000 (s). Experimental verification of these quantities that describe the levels of all these molecular concentrations can be very difficult if not impossible and they relate indirectly to the ultimate consequence of PDT for cell killing. In [5] we introduced a cell killing model that related the molecular concentrations of the singlet oxygen and the unoxidized receptors to the cell survival ratio, which can be measured with an in vitro cell model. The same software (MATLAB) is used for producing the simulations for the decision mechanism of a single cell model design. The output of the time dependent Blahut Arimoto algorithm is the cell survival probability (Figure 3). The distortion measure *d *that quantifies how disadvantageous a decision y is, in response to the stimulus vector $x=\left(x_{1},x_{2},x_{3}\right)$ is defined by the equation $d\left(\left(x_{1},\;x_{2},\;x_{3}|y=survival\right)\right)=10$ if $x_{i}\geq x_{i}^{th}$ for some i, and $d\left(\left(x_{1},\;x_{2},\;x_{3}|y=death\right)\right)=10^{-1}$ if $x_{i}\geq x_{i}^{th}$ for some i, and a small number otherwise. The thresholds $x_{i}^{th}$ for the normalized concentrations were all set to 0.5. This distortion measure penalizes a cell survival error more than cell death error for given stimuli, by one order of magnitude. For the range of the Lagrange multipliers the equation *s *= -*e*^-*n *^was used and in the simulations n varied over a finite set of integers (a sample of n values from 1 to 20 was taken for the simulations below). The initial survival probability $q^{0}\left(y=surv\right)=1-q_{0}$ was set equal to 0.9. The treatment parameters for the PDT model that was introduced in our previous work [5], was linked to the input of this algorithm.

## Discussions

The effort to link biochemical pathways and molecular interactions to the behavior of whole cells and to infer causality from statistical correlation in large data sets in photo-chemotherapy is a matter of considerable difficulty, and to account for all biological variation is a very challenging goal. The existence of more than one PDT tissue destruction mechanism in vivo for the treatment of intraocular retinoblastoma like tumor, has been suggested and documented in [31] where an early direct cell damage was followed by a subsequent late damage occurring in the tumor tissue left in situ after treatment, resulting in a biphasic pattern in the cell survival curve as a function of time. In [32], experiments on Chinese hamster cells with phthalocyanine dyes and split light fluence indicated that cells can repair sublethal photo cytotoxic damage during the course of several hours. Although direct cytotoxicity to the tumor cells has been shown to be relatively small after PDT and to increase with time after treatment [33], examples of in vitro mammalian cell curves as functions of exposure time for different photosensitizer concentrations show that for an acute high dose treatment (vast majority of PDT treatments) the cell survival ratio decreases to less than 1% in the course of a few minutes.

Discrepancies may be due to many factors such as light attenuation passing through the skin resulting in a relatively lower energy dose to some cells than others or the fact that the tumor vasculature is a primary target of PDT. The local micro-environment might have significant impact on PDT response. Vascular effects can be secondary to cell death or conversely, cell death can be secondary to vascular shutdown. Another factor that might affect the final outcome is the triggering of the immune responses, local or systemic.

According to Langton, the 'logical form' of an organism can be separated from its material basis of construction, and that 'aliveness' will be found to be a property of the former, not of the latter [34]. It is the major assumption in the field of Artificial Life (AI) that life is a property of the organization of matter, rather than a property of the matter itself. Organization reduces uncertainty through a process of information collection, management and use. A conceptual and mechanistic system biology mathematical model that is based on information theory can yield valuable insights since cellular behavior cannot be summarized in population averages [35]. The Blahut Arimoto model has several features that are consistent with the experimental results. For the parameter s, estimation can be performed using experimental data, and a range of values can be recovered. The shapes of the survival curves and their correlation with the parameter s will depend on the structure of the rate equations, the type of cell decision algorithm adopted and the accuracy of the experimental data. Different values of the parameters will be predictive of different model curve topologies. Although the origin of the cell parameter s in a cell population for this biochemical model remains non-identifiable from the biophysics point of view, high-likelihood predictions can still be made by appropriate choice and calibration of this parameter. The survival probability predicted by the rate distortion function and calculated by the Blahut Arimoto algorithm, and the variability in the graphs resulting from different values of the parameters provide a framework for the interpretation of self-renewal capabilities of the cell and its ability to generate drug resistance.

The model presented in this report is applicable to the study of cell killing mechanisms in other cases such as hydrogen peroxide *H*_2_*O*_2 _induced cell death in neurodegenerative diseases. Neuronal death observed in neurodegenerative disorders has been shown to be related to free radical damage and the mechanisms by which reactive oxygen species may damage or kill neurons have been investigated, with a series of experiments designed to document events associated with *H*_2_*O*_2 _induced cell death in primary neuronal culture [36]. Moreover, this model provides a conceptual frame for the study of hyperthermia induced cell death. Hyperthermia also induces apoptosis in a wide range of cancer cells [37]. The way hyperthermia initiates the intrinsic pathway of apoptosis is yet not completely elucidated, but it is known to involve the transmission of the temperature elevation signal to the mitochondrion through proteins belonging to the Bcl-2 family. Recent preclinical developments show the importance of heat shock proteins and other proteins interfering and regulating the intrinsic and extrinsic pathways of apoptosis [38]. It has been suggested that intracellular de novo synthesis and polymerization of both RNA- and DNA-molecules as well as protein synthesis are decreased in vitro at temperatures between 42 and 45°C in a dose dependent manner. Whereas RNA- and protein synthesis recover rapidly after termination of heat exposure, DNA-synthesis is inhibited for a longer period [39,40]. The heat shock induces an aggregation of denatured proteins at the nuclear matrix owed to the insolubility of cellular proteins after heat-induced protein unfolding, entailing an enhancement of the nuclear protein concentration. Increase of the nuclear protein content by heat consequently affects several molecular functions (including DNA-synthesis and -repair) when a certain thermal dose is exceeded. A variation of the threshold dose among distinct cell lines is to be expected. Cells are surrounded by electromagnetic fields and the ion distributions inside and outside the cells are also at different concentrations depending on their charge and their type (healthy cells or tumor cells). Heat is transported by means of the extracellular space, ionically bound, to the intracellular space. After the compensation capacity of the cell is exceeded, it is natural to expect that a very small change in the temperature within and outside the cell membrane will be sufficient to affect, or block the metabolic processes or even to denature the proteins (Celsius 42+, Koln, Germany, modalities and procedural technologies for clinical hyperthermia). Now, heat is not a property of a system or body, but instead is always associated with a process of some kind, and is synonymous with heat flow and heat transfer. It has the characteristic feature that it increases the entropy. According to Shannon's theory, entropy measures the information contained in a message (entropy is often used as a characterization of the information content of a data source). Assuming that heat increases the conformational entropy which implies the thermic denaturation of proteins and that heat shock proteins down regulate antioxidants and therefore up regulate oxidative stress as demonstrated by several experimental studies, leading to lipid peroxidation of the lipid components, a model similar to one presented above for PDT can be derived for making binary cell fate decisions (death/survival), as a result of heat exposure (hyperthermia). There are some obvious similarities with PDT such that heat toxicity is the result of oxidative stress. A computational model that will be based on information theory (information enters the cell in this case in the form of increasing entropy, a word with informational context "disorganize") can be used not only to quantitatively describe how the heat shock signal is transformed to a cell death probability (the activation of the thermal signal processing occurring when any of some predefined molecular stimulants increases higher than a predefined threshold) but also to elucidate the main factors causing cell death after heat exposure from a qualitative viewpoint.

Information implies both facts and transmission of facts. The definition of information is content neutral and in Shannon's distortion theory, information is interpreted as what reduces uncertainty. It also presupposes knowledge of a priori probabilities. These probabilities need to be designed or calculated in a way that they will reflect the varieties of environmental stimuli. It is important to decipher the meaning of information available to a cell as something that determines its activity. Information has no mass, energy, or spatiotemporal extension. Nevertheless it is a distinct, objective entity. This entity can be traced through detectable differences. For example, the cell, as an information system has the ability to discriminate and select between cell fates (which is what we call cell decision making). In fact, the manifestation of information can be found in the existence of alphabets (where as alphabet we interpret the set of physical states that can be realized in some system), the combination of codes (where as a code we consider a collection of the letters of alphabets that follow some pattern-words) and the variety of codes that determine the state of the system.

In the work of James G. Miller [41] on living systems, it was postulated that by the information input of its charter or genetic input, or by changes in behavior brought about by rewards and punishments from its suprasystem, a system develops a preferential hierarchy of values that gives rise to decision rules which determine its preference for one internal steady-state value rather than another. This was defined as the purpose of the system, which will also have an external goal related to its purpose. Therefore the goal of the system is determined by a system on a higher level. This was confirmed in the study of a cell model in the work of Perkins and Swain [42] who characterized cellular decision-making as having three main tasks: a cell must (1) estimate the state of its environment by sensing stimuli; (2) make a decision informed by the consequences of the alternatives; and (3) perform these functions in a way that maximizes the fitness of the population. Porter and Iglesias suggested the distortion theory framework, providing a complementary perspective on decision-making, regarding these three tasks as a single process. According to Porter and Iglesias [8], the distortion measure *d *: defines accurate sensing (task 1) by how heavily it penalizes small mistakes, and it quantifies the disadvantages of alternative decisions (task 2); the expected distortion describes how accurate sensing must be (task 1) and how much disadvantage the cell can afford in making a decision (task 2); the resulting optimal strategies fulfill task 3 by making choices with decisiveness proportional to the information available. The existence of suprasystem that determines the goal of the system of a cell (which can be either the tumor cell population for tumor growth or the healthy tissue surrounding the cell performing regulatory functions such as immune dynamics, angiogenesis, etc. or an unidentified entity) is reflected on the structure of the distortion measure. The condition of an "observer" distinguishable from the system that determines the goal of the system is a prerequisite for the definition of information in cybernetics by Wiener, which is founded on the issues of control and communication.

A generalization of the concept of mutual information used in this report is the interaction information. The "interaction information" [43] is a generalization of the mutual information, and expresses the amount information (redundancy or synergy) bound up in a set of variables, beyond that which is present in any subset of those variables:

$$Q\left(X,Y,Z\right)=I\left(X,Y|Z\right)-I\left(X,Y\right)=\underset{x,y,z}{\sum }P\left(x,y,z\right)\cdot \text{log}\frac{P\left(x,y,z\right)\cdot P\left(x\right)\cdot P\left(y\right)\cdot P\left(z\right)}{P\left(x,y\right)\cdot P\left(y,z\right)\cdot P\left(z,x\right)}$$

Q measures associations between variables, and not the direction of the transmission: 'This means that nothing is gained formally by distinguishing transmitters from receivers, therefore it goes beyond the Shannon framework of linear transmissions [44]. An interaction is regularity, a pattern, a dependence present only in the whole set of events, but not in any subset. It is symmetric and undirected, so directionality no longer needs to be explained by, e.g. causality [45]. Positive interaction implies synergy, and variables has been associated with the non-separability of a system in quantum physics [46] and with the origin of synergy in relationships between neurons [47]. Q measures the amount of influence on the relationship between × and Y, resulting from the introduction of Z [45]. It is the amount of information that is common to all variables but not present in any subset. The interaction information may provide the appropriate framework for the study of the Radiation induced Bystander effect (unirradiated cells exhibit irradiated effects as a result of signals received from nearby irradiated cells), a well-established consequence of exposure of living cells to radiation [48]. Although cell to cell communications in normal and carcinogenic cells have been discussed extensively [49,50], and it is believed that in general cell to cell regulatory signals are conducted by chemical and electrical signals (Gap Junctional Intercellular Communication (GJIC) or Distant Signaling Intercellular Communication (DSIC)) [50], it remains an assumption that these signals are propagated by a Brownian diffusive motion, because this yields to relatively satisfactory results in simulations of bystander effects [50]. To understand the bystander synergistic effect in the case of radiation, we observe that if *X *is a cell state cell (target cell), and *Y *is some parameter that represents radiation, and *Z *is a cell state (neighbor cell), *Q*(*X, Y, Z*) can be understood as the difference between the decrease in entropy of *Z *achieved by the joint attribute *XY *(cell irradiation event) and the expected decrease in entropy with the assumption of independence between *X *and *Y*.

## Conclusions

In this study a model of a cell decision mechanism is proposed, which captures certain observed characteristics of a cell behavior during photo-irradiation and pharmacological treatment (Type II PDT) using rate distortion theory to quantify the goals of a binary decision process (cell survival - cell death). The main components of the model are, the time dependent distribution of molecular stimuli, the distortion function (or measure), the conditional probability of the cell decision strategy, the cell survival probability, the expected distortion and the rate distortion function which quantifies a limit on how well the goals can be achieved given the stimulation. The results are independent of the biological mechanism by which the cell strategy is implemented and the Blahut Arimoto algorithm is used to derive optimal pathways. The model requires knowing the probability distribution of the stimuli as its input. For a variety of Lagrange multipliers, there is a corresponding variety of optimal pathways, but an approximation of the distortion function around which the pathway is optimized, is possible, based on algebraic properties of the algorithm (the distortion constraint) and numerical and experimental data [8]. Intracellular molecular interactions can be studied with the purpose of extracting useful conclusions, by using computational methods. In this report we used a previous developed systems biology model that includes detailed molecular pathways induced by PDT treatment leading to cell death, which we coupled to a cell decision making algorithm that is based on the mutual information between cell death stimulation and cell response as the output of a bio molecular communication channel. This line of research can be relevant to future improvement and management of cancer treatment methodologies. The cell survival probability is modeled as the output of an optimization process of transmitting the death signal through a communication channel with a possible environmental and/or inherent distortion. Modeling results can be compared directly to experimental results that are based on the levels of measurable molecular concentrations and cell survival ratios, for optimization of the unknown parameters or/and for design of different in vitro studies of PDT. This modeling establishes a framework that may also be able to address questions such as why do cell types, although they share the same genome, they are in general stable entities represented by different observable cell fates with certain rules that govern their molecular dynamics and do not gradually change into other forms.

## Competing interests

The author declares that he has no competing interests.

## Declarations section

The publication costs for this article were funded by the corresponding author, Ioannis Gkigkitzis.

This article has been published as part of *BMC Medical Genomics *Volume 6 Supplement 3, 2013: Selected articles from the IEEE International Conference on Bioinformatics and Biomedicine 2012: Medical Genomics. The full contents of the supplement are available online at http://www.biomedcentral.com/bmcmedgenomics/supplements/6/S3.

## References

[B1] Sharman WM Allen CM van Lier JE 'Role of activated oxygen species in photodynamic therapy' Methods in enzymology 2000 6 376 400 10907528 10.1016/s0076-6879(00)19037-8

[B2] Buytaert E Callewaert G Vandenheede JR Agostinis P 'Deficiency in apoptotic effectors Bax and Bak reveals an autophagic cell death pathway initiated by photodamage to the endoplasmic reticulum' Autophagy 2006 6 3 238 240 16874066 10.4161/auto.2730

[B3] Albeck JG Burke JM Spencer SL Lauffenburger DA Sorger PK 'Modeling a snap-action, variable-delay switch controlling extrinsic cell death' PLoS biology 2008 6 12 2831 2852 19053173 10.1371/journal.pbio.0060299 PMC2592357

[B4] Tyson JJ Baumann WT Chen C Verdugo A Tavassoly I Wang Y Weiner LM Clarke R 'Dynamic modelling of oestrogen signalling and cell fate in breast cancer cells' Nat Rev Cancer 2011 6 7 523 532 10.1038/nrc3081 21677677 PMC3294292

[B5] Gkigkitzis I Feng Y Yang C Lu JQ Hu XH 'Modeling of Oxygen Transport and Cell Killing in Type-II Photodynamic Therapy' Photochem Photobiol 2012 6 4 969 977 10.1111/j.1751-1097.2012.01145.x 22443292

[B6] Howland JL 'Introduction to cell physiology: information and control' 1968 Macmillan 1968

[B7] Gkigkitzis I Hu X-H 'A model of cellular decision making in photodynamic therapy of cancer' Bioinformatics and Biomedicine (BIBM), 2012 IEEE International Conference on: 4-7 October 2012 2012 1 5 10.1109/BIBM.2012.6392704

[B8] Porter JR Andrews BW Iglesias PA 'A framework for designing and analyzing binary decision-making strategies in cellular systems' Integr Biol (Camb) 2012 6 3 310 317 10.1039/c2ib00114d 22370552 PMC4547352

[B9] Andrews BW Iglesias PA 'An information-theoretic characterization of the optimal gradient sensing response of cells' PLoS computational biology 2007 6 8 e153 10.1371/journal.pcbi.0030153 17676949 PMC1937015

[B10] Wallace R 'A rate distortion approach to protein symmetry' Biosystems 2010 6 2 97 108 10.1016/j.biosystems.2010.05.002 20553799

[B11] Von Neumann J Burks AW 'Theory of self-reproducing automata' 1966 University of Illinois Press 1966

[B12] Denton M 'Evolution : a theory in crisis' 1986 Adler & Adler 1st U.S. edn. 1986

[B13] Gougeon ML Kroemer G 'Charming to death: caspase-dependent or -independent?' Cell death and differentiation 2003 6 3 390 392 10.1038/sj.cdd.4401199 12700640

[B14] Luo Y Kessel D 'Initiation of apoptosis versus necrosis by photodynamic therapy with chloroaluminum phthalocyanine' Photochemistry and photobiology 1997 6 4 479 483 10.1111/j.1751-1097.1997.tb03176.x 9337618

[B15] Blahut RE 'Computation of Channel Capacity and Rate-Distortion Functions' Ieee T Inform Theory 1972 6 4 4601

[B16] Berger T 'Rate distortion theory; a mathematical basis for data compression' 1971 Prentice-Hall 1971

[B17] Ledder G Logan JD Joern A 'Dynamic energy budget models with size-dependent hazard rates' J Math Biol 2004 6 6 605 622 10.1007/s00285-003-0263-1 15164225

[B18] Burns RC Lawson AD 'Quantized Probability Circuit Design Principles Applied to Linear Circuits' Ieee T Reliab 1964 6 2 16 &

[B19] Lomsadze YM 'Relativistically Invariant Formulation of Theory of Quantized Probability Amplitude Field' Nucl Phys 1962 6 1 147 &

[B20] Link G ebrary Inc 'One hundred years of Russell's paradox mathematics, logic, philosophy'. 'Book One hundred years of Russell's paradox mathematics, logic, philosophy' 2004 6 Walter de Gruyter 662 (Ed.)^(Eds.)

[B21] Van Heijenoort J 'From Frege to Gödel; a source book in mathematical logic, 1879-1931 1967 Harvard University Press 1967

[B22] Simmons K 'Sets, classes and extensions: A singularity approach to Russell's paradox' Philos Stud 2000 6 2 109 149 10.1023/A:1018666804035

[B23] von Neumann J 'An axiomatization of set theory' Aut Aut 1997 6 107 123

[B24] Priest G 'Beyond the limits of thought' 2002 Clarendon/Oxford University Press 2002

[B25] Chesson P 'Predator-Prey Theory and Variability' Annu Rev Ecol Syst 1978 6 323 347 10.1146/annurev.es.09.110178.001543

[B26] Perthame B Gauduchon M 'Survival thresholds and mortality rates in adaptive dynamics: conciliating deterministic and stochastic simulations' Math Med Biol 2010 6 3 195 210 10.1093/imammb/dqp018 19734200

[B27] Masutani K 'Effects of survival thresholds upon one-dimensional dynamics of single-species populations' B Math Biol 1993 6 1 1 13

[B28] Graf S Luschgy H Pages G 'The Local Quantization Behavior of Absolutely Continuous Probabilities' Ann Probab 2012 6 4 1795 1828 10.1214/11-AOP663

[B29] Gray RM Neuhoff DL 'Quantization' Ieee T Inform Theory 1998 6 6 2325 2383 10.1109/18.720541

[B30] Tyson JJ Novak B 'Functional Motifs in Biochemical Reaction Networks' Annu Rev Phys Chem 2010 6 219 240 10.1146/annurev.physchem.012809.103457 20055671 PMC3773234

[B31] Winther J 'Photodynamic therapy effect in an intraocular retinoblastoma-like tumour assessed by an in vivo to in vitro colony forming assay' British journal of cancer 1989 6 6 869 872 10.1038/bjc.1989.184 2525401 PMC2246719

[B32] Ben-Hur E Rosenthal I Leznoff CC 'Recovery of Chinese hamster cells following photosensitization by zinc tetrahydroxyphthalocyanine' Journal of photochemistry and photobiology B, Biology 1988 6 2 243 252 10.1016/1011-1344(88)80007-1 3149991

[B33] Selman SH Kreimer-Birnbaum M Chaudhuri K Garbo GM Seaman DA Keck RW Ben-Hur E Rosenthal I 'Photodynamic treatment of transplantable bladder tumors in rodents after pretreatment with chloroaluminum tetrasulfophthalocyanine' The Journal of urology 1986 6 1 141 145 3712601 10.1016/s0022-5347(17)44759-8

[B34] Langton CG Center for Nonlinear Studies (Los Alamos National Laboratory) Santa Fe Institute (Santa Fe N.M.) Apple Computer Inc 'Artificial life : the proceedings of an interdisciplinary workshop on the synthesis and simulation of living systems, held September, 1987 in Los Alamos, New Mexico' 1989 Addison-Wesley Pub. Co. 1989

[B35] Gunawardena J 'Signals and systems: Towards a systems biology of signal transduction' P Ieee 2008 6 8 1386 1397

[B36] Whittemore ER Loo DT Watt JA Cotman CW 'A detailed analysis of hydrogen peroxide-induced cell death in primary neuronal culture' Neuroscience 1995 6 4 921 932 10.1016/0306-4522(95)00108-U 7675214

[B37] Rong Y Mack P 'Apoptosis induced by hyperthermia in Dunn osteosarcoma cell line in vitro' Int J Hyperthermia 2000 6 1 19 27 10.1080/026567300285394 10669314

[B38] Beere HM 'Death versus survival: functional interaction between the apoptotic and stress-inducible heat shock protein pathways' The Journal of clinical investigation 2005 6 10 2633 2639 10.1172/JCI26471 16200196 PMC1236700

[B39] Streffer C 'Aspects of metabolic change after hyperthermia' Recent results in cancer research Fortschritte der Krebsforschung Progres dans les recherches sur le cancer 1988 6 7 16 10.1007/978-3-642-83260-4_2 3287523

[B40] Falk MH Issels RD 'Hyperthermia in oncology' Int J Hyperthermia 2001 6 1 1 18 11212876 10.1080/02656730150201552

[B41] Miller JG 'Living systems' 1995 University Press of Colorado 1995

[B42] Perkins TJ Swain PS 'Strategies for cellular decision-making' Molecular systems biology 2009 6 326 19920811 10.1038/msb.2009.83 PMC2795477

[B43] McGill WJ 'Multivariate Information Transmission' Psychometrika 1954 6 2 97 116 10.1007/BF02289159

[B44] Leydesdorff L 'Interaction information: linear and nonlinear interpretations' Int J Gen Syst 2009 6 6 681 685 10.1080/03081070902993038

[B45] Jakulin A Bratko I 'Analyzing attribute dependencies' Lect Notes Artif Int 2003 6 229 240

[B46] Cerf NJ Adami C 'Entropic Bell inequalities' Phys Rev A 1997 6 5 3371 3374 10.1103/PhysRevA.55.3371

[B47] Brenner N Strong SP Koberle R Bialek W van Steveninck RRD 'Synergy in a neural code' Neural Comput 2000 6 7 1531 1552 10.1162/089976600300015259 10935917

[B48] Lorimore SA Wright EG 'Radiation-induced genomic instability and bystander effects: related inflammatory-type responses to radiation-induced stress and injury? A review' International journal of radiation biology 2003 6 1 15 25 12556327

[B49] Trosko JE Ruch RJ 'Cell-cell communication in carcinogenesis' Frontiers in bioscience : a journal and virtual library 1998 6 d208 236 9458335 10.2741/a275

[B50] Nikjoo H Khvostunov IK 'A theoretical approach to the role and critical issues associated with bystander effect in risk estimation' Human & experimental toxicology 2004 6 2 81 86 10.1191/0960327104ht422oa 15070065

